# Coordination of AMPK and YAP by *Spatholobi Caulis* and Procyanidin B2 Provides Antioxidant Effects In Vitro and In Vivo

**DOI:** 10.3390/ijms232213730

**Published:** 2022-11-08

**Authors:** Su-Jin Bae, Seon Been Bak, Young Woo Kim

**Affiliations:** School of Korean Medicine, Dongguk University, Gyeongju 38066, Korea

**Keywords:** AMPK, YAP, *Spatholobi Caulis*, liver injury, procyanidin B2, acetaminophen

## Abstract

The liver is vulnerable to oxidative attacks from heavy metals, such as iron, as well as some drugs, including acetaminophen. It has been shown that enhanced oxidative stress in the liver leads to excessive ROS production and mitochondrial dysfunction, resulting in organ injury. The beneficial effects of *Spatholobi Caulis* (SC), a natural herbal medicine, include treating ischemic stroke, inhibiting tumor cell invasion, pro-angiogenic activities, and anti-inflammatory properties. Scientific studies on its effects against hepatotoxic reagents (e.g., iron and acetaminophen), as well as their underlying mechanisms, are insufficient. This study examined the antioxidant effects and mechanisms of SC in vitro and in vivo. In cells, the proinflammatory mediator, arachidonic acid (AA), plus iron, significantly induced an increase in ROS generation, the damage in mitochondrial membrane potential, and the resulting apoptosis, which were markedly blocked by SC. More importantly, SC affected the activation of AMP-activated protein kinase (AMPK)-related proteins, which were vital to regulating oxidative stress in cells. In addition, SC mediated the expression of Yes-associated protein (YAP)-related proteins. Among the active compounds in SC, the procyanidin B2, but not liquiritigenin, daidzein, and genistein, significantly inhibited the cytotoxicity induced by AA + iron, and activated the LKB1-AMPK pathway. In mice, the oral administration of SC alleviated the elevations of ALT and histological changes by the acetaminophen-induced liver injury. These results reveal the potential of SC and a key bioactive component, procyanidin B2, as antioxidant candidates for hepatoprotection.

## 1. Introduction

Oxidative stress contributes to the progression of various diseases in humans [1,2]. In particular, chronic liver damage caused by oxidative stress can progress to hepatitis, liver fibrosis, and liver cancer [3,4]. Oxidative stress results in the excessive production of oxidative free radicals and reactive oxygen species (ROS) [5]. ROS generated in the normal aerobic metabolism perform intracellular signaling molecules in various biological processes [6]. In addition, it enhances the immunologic defense against bacteria and other pathogens [7]. On the other hand, the excessive production of ROS sometimes leads to cellular dysfunction, including mitochondrial DNA mutations, hyperactivation of inflammatory responses, and inflammation [8].

AMP-activated protein kinase (AMPK) is an essential enzyme in regulating cellular energy homeostasis. It is activated by increased intracellular AMP and changes in the ADP/ATP ratios, such as hypoglycemia, hypoxia, high-intensity exercise, and ischemia [9]. The activation of AMPK enhances glucose uptake and inhibits gluconeogenesis. In addition, it promotes fatty acid oxidation and inhibits fat synthesis and cholesterol synthesis [10]. Therefore, it is expected to be important in preventing and treating metabolic diseases, such as obesity and type 2 diabetes [11]. Moreover, the activation of AMPK has been reported to protect cardiac muscle cells, osteoblastic cells, nerve cells, and liver cells [12,13,14,15].

The Hippo–Yes-associated protein (YAP) pathway is a kinase cascade that regulates vital cellular functions; in this pathway, the YAP is a key transcriptional regulator [16]. In previous studies, the activation of AMPK and inhibition of YAP indicated hepatocellular protection, suggesting that the AMPK pathway and Hippo–YAP are connected [17]. It has been reported that YAP attenuated acetaminophen-induced hepatotoxicity [18]. A previous study reported that regulating AMPK signaling and YAP pathways attenuated CCl4-induced acute liver injury in mice [19].

*Spatholobi Caulis* (SC), a dried vine of *Spatholobus suberectus* Dunn included in Leguminosae, has reported effects on the treatment of ischemic stroke, inhibition of tumor cell invasion and pro-angiogenic activity, and anti-hepatitis C virus activity [20,21,22,23]. In addition, SC can potentially prevent deep vein thrombosis through anti-inflammation by promoting SIRT1 and Nrf2 expression [24], and induce an increase in anti-obesity by upregulation of the AMPK pathway in mice [25]. On the other hand, there have been few studies on the hepatoprotective effects of SC and its mechanism.

Based on the reported studies, it was hypothesized that SC would protect hepatocytes by inhibiting oxidative stress, possibly related to the activation of AMPK-related signaling. This study examined the protective effect of SC against cytotoxicity induced by arachidonic acid (AA) + iron treatment in vitro and acute acetaminophen (APAP)-induced hepatotoxicity in vivo. In addition, the signaling pathway affecting the antioxidant effects of SC was demonstrated, and the hepatoprotective effects of a critical bioactive component of SC were verified.

## 2. Results

### 2.1. SC Protects Hepatocytes against AA + Iron-Induced Cytotoxicity

**Figure 1:**
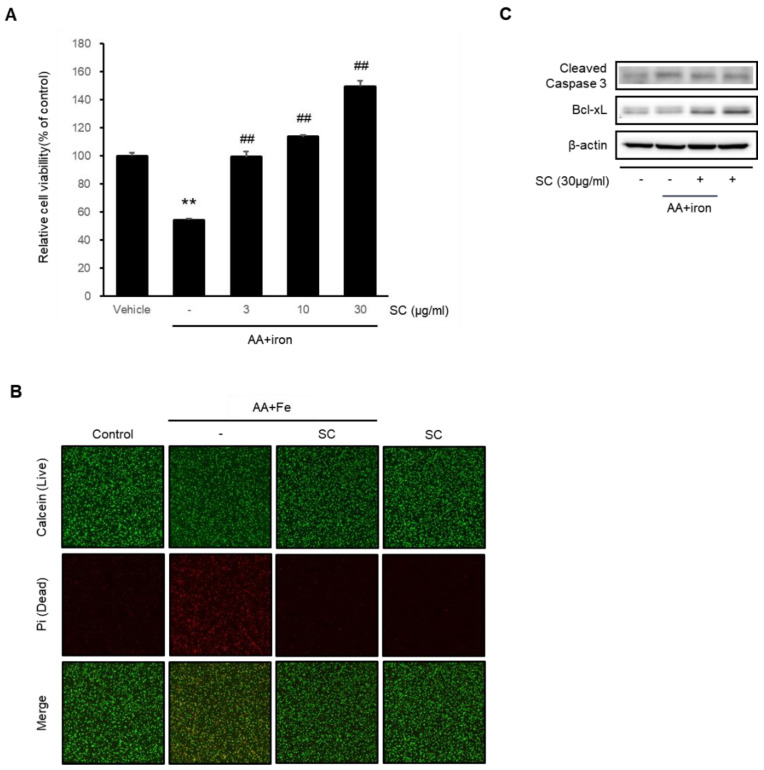
Protective effect of SC on cytotoxicity caused by AA + iron. (**A**) Cell viability was assessed by an MTT assay. HepG2 cells were treated with SC (3, 10, and 30 μg/mL) for 1 h and incubated with 10 μM AA for 12 h, followed by incubation with 5 μM iron for 6 h. The data represent the mean ± SD of three replicates (** *p* < 0.01 between control and AA + iron-treated cells; ## *p* < 0.01 between AA + iron and AA + iron-treated cells with SC). (**B**) The cell viability was measured using calcein AM and propidium iodide (Pi) staining. HepG2 cells were incubated with AA + iron as (**A**), with or without SC 30 μg/mL, and then stained with calcein AM (0.5 μg/mL) and Pi (0.5 μg/mL). Calcein and Pi fluorescence was detected using a fluorescent microscope (×100); (**C**) immunoblotting analysis for apoptosis-related proteins was performed with HepG2 cell lysates. HepG2 cells were stimulated by AA with or without SC 30 μg/mL, and then the iron for 1 h.

An MTT assay was performed to evaluate the cytoprotective effect of SC. AA + iron-induced oxidative stress in HepG2 cells was treated with SC (3, 10, and 30 μg/mL). AA + iron-reduced cell viability significantly compared to the control group. On the other hand, the cell viability increased significantly in a dose-dependent manner when the cells exposed to AA + iron were treated with SC (Figure 1A). The highest cell viability was observed in the cells treated with 30 μg/mL of SC. This concentration was used in the following experiments. The cytoprotective effects of SC against AA + iron-induced damage were verified by calcein AM and Pi staining (Figure 1B). The levels of cleaved caspase-3 and Bcl-xL were measured by immunoblotting analysis to investigate further the protective effect of SC against AA + iron-induced cytotoxicity (Figure 1C). The AA + iron treatment increased the levels of cleaved caspase-3 and Bcl-xL, whereas the SC treatment prevented this effect.

### 2.2. SC Inhibits AA + Iron-Induced ROS Generation and Mitochondrial Dysfunction

**Figure 2:**
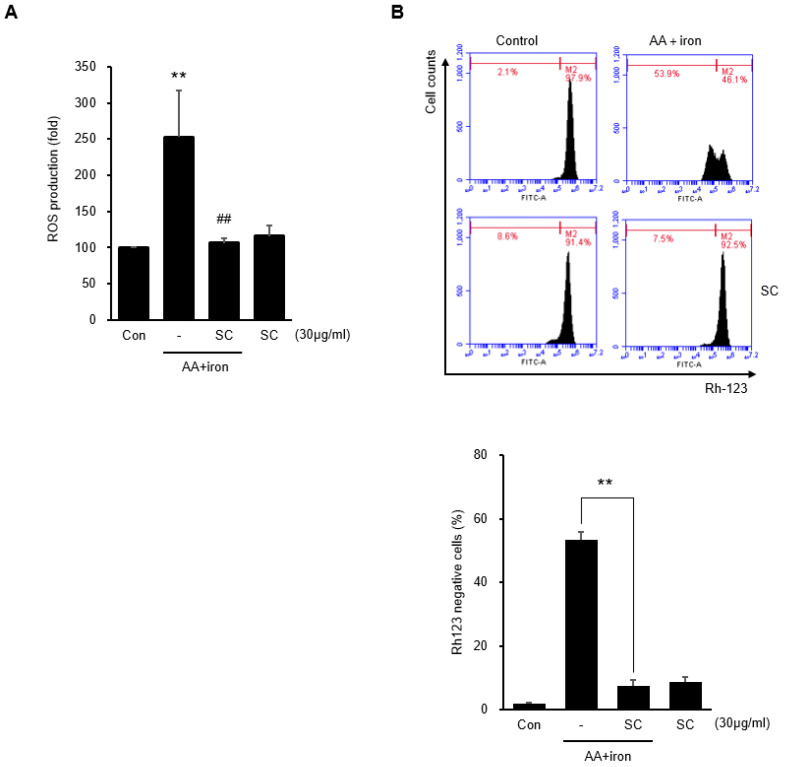
Effect of SC on AA + iron-induced ROS generation and mitochondrial dysfunction. HepG2 cells were treated, as described in Figure 1C. (**A**) Cellular ROS production was measured by DCFH-DA; (**B**) mitochondrial membrane permeability (MMP) was measured by flow cytometry. The cells were treated as described in Figure 1C and stained with rhodamine 123 (0.05 μg/mL) for 1 h. All data represent the mean ± SD (** *p* < 0.01 between control and AA + iron-treated cells; ## *p* < 0.01 between AA + iron and AA + iron-treated cells with SC).

The intracellular ROS levels were measured to determine the antioxidant effect of SC. AA + iron increased intracellular ROS generation significantly, whereas 30 μg/mL SC alone did not increase intracellular ROS generation. Pretreatment with 30 μg/mL SC inhibited intracellular ROS generation by AA + iron (Figure 2A). Rhodamine 123, which selectively accumulates in the mitochondrial matrix based on the mitochondrial membrane permeability, was used with flow cytometry to evaluate the mitochondrial dysfunction. AA + iron significantly increased rhodamine 123 negative cells compared to the control, but 30 μg/mL SC alone did not produce significant changes. Pretreatment with 30 μg/mL SC inhibited rhodamine 123 negative cells caused by AA + iron (Figure 2B). These results suggest that SC inhibits AA + iron-induced mitochondrial dysfunction and eventually protects hepatocytes.

### 2.3. SC Activates AMPKα

**Figure 3:**
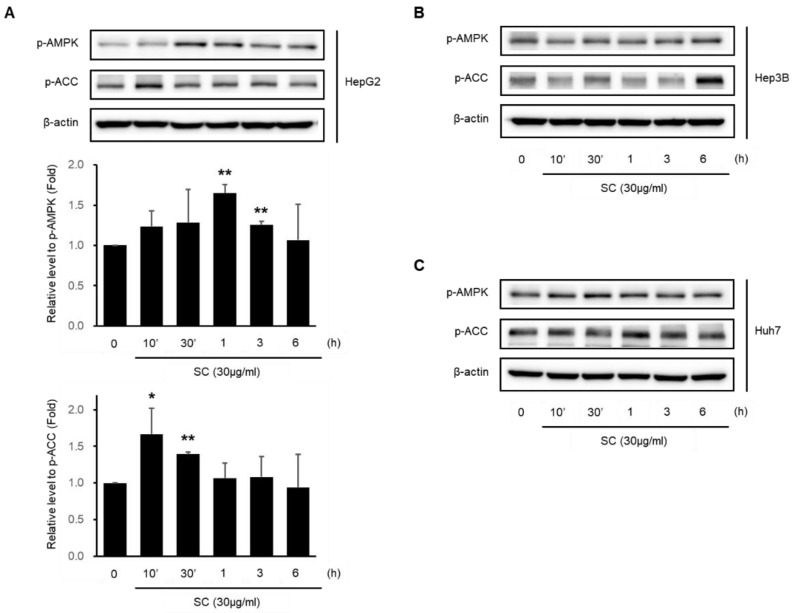
Effect of SC on AMPK activation. Immunoblotting analysis for key signaling proteins of the AMPK signaling pathway was performed with HepG2 (**A**); Hep3B (**B**); and Huh-7 (**C**) cell lysates. The cells were incubated in serum-free media for 12 h, followed by treatment with 30 μg/mL SC for the indicated time. Expression of p-AMPK and p-ACC in HepG2 represents the mean ± SD (* *p* < 0.05, ** *p* < 0.01 between control and SC-treated cells).

AMPK activation was verified by immunoblotting analysis to investigate the mechanism for the effect of SC. SC (30 μg/mL) induced the phosphorylation of AMPK in HepG2 (Figure 3A), Hep3B (Figure 3B), and Huh7 (Figure 3C) cells. In particular, the maximum activation of AMPK was observed at 1–3 h in HepG2 cells. ACC, which is known as a major downstream target of AMPK, was also phosphorylated by the SC treatment in HepG2 (Figure 3A), Hep3B (Figure 3B), and Huh7 (Figure 3C) cells. In HepG2 cells, the maximum phosphorylation of ACC was observed at 10–30 min.

### 2.4. SC Protects Hepatocytes via Activation of the LKB1-AMPK Pathway

**Figure 4:**
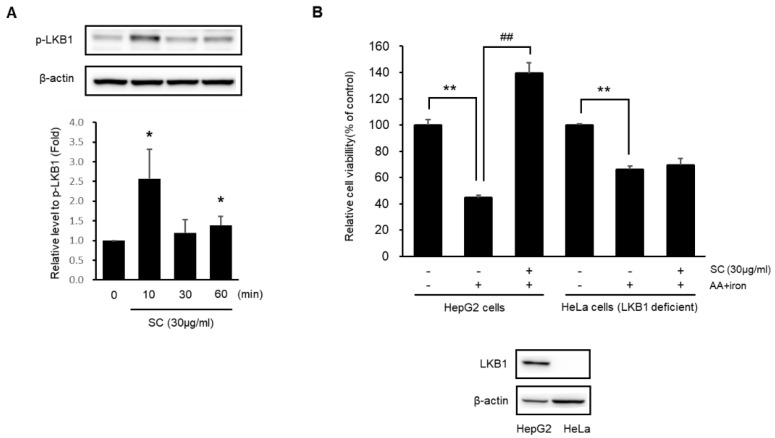
Effect of SC on LKB1 activation. (**A**) Immunoblotting analysis of p-LKB1 was performed with HepG2 cell lysates. The cells were treated as described in Figure 3A, and incubated for the indicated time. Expression of p-LKB1 represents the mean ± SD of three replicates (* *p* < 0.05 between control and SC-treated cells). (**B**) Effect of SC on the cytotoxicity caused by AA + iron on HepG2 and LKB1-deficient HeLa cells. HepG2 and HeLa Cells were incubated as in Figure 1A. After incubation, an MTT assay was performed. Data represent the mean ± SD (** *p* < 0.01 between control and AA + iron-treated cells; ## *p* < 0.01 between AA + iron and AA + iron-treated cells with SC).

Experiments were conducted to determine if SC has a cytoprotective effect by activating the LKB1-AMPK pathway. SC (30 μg/mL) induced the phosphorylation of LKB1, a major upstream target of AMPK (Figure 4A). SC inhibited AA + iron-induced apoptosis in HepG2 cells but not LKB1-deficient HeLa cells (Figure 4B). These results suggest that SC protects against AA + iron-induced cytotoxicity by activating the LKB1-AMPK pathway.

### 2.5. SC Activates the Hippo–YAP Pathway

**Figure 5:**
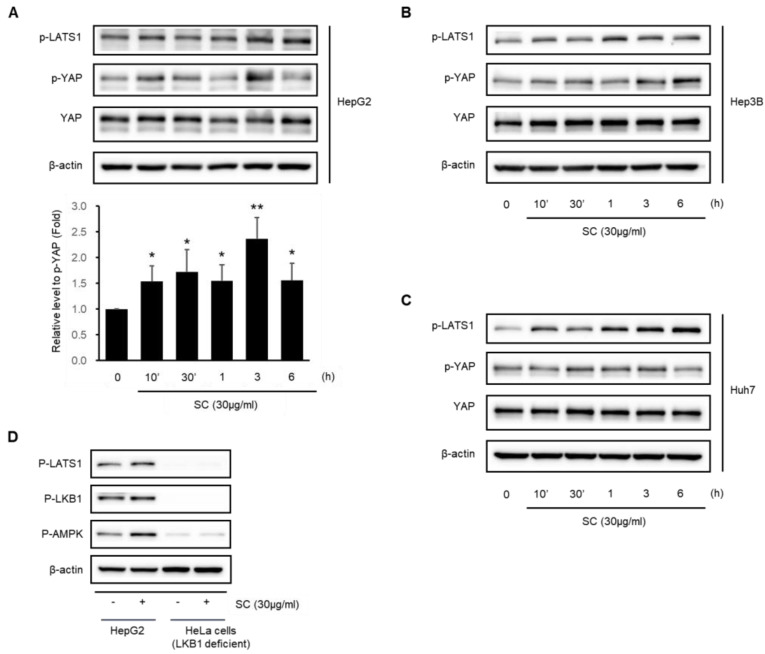
Effects of SC on YAP activation. Immunoblotting analysis of YAP-associated proteins was performed with HepG2 (**A**); Hep3B (**B**); and Huh-7 (**C**) cell lysates. The lysates of cells progressed as described in Figure 3. Expression of p-YAP in HepG2 represents the mean ± SD (* *p* < 0.05, ** *p* < 0.01 between the control and SC-treated cells). (**D**) Comparison of p-LATS1, p-LKB1, and p-AMPK expression in LKB1-deficient HeLa and HepG2 cells. The cells were incubated as in Figure 3 and treated with 30 μg/mL SC for 1 h.

This study examined whether SC mediates the Hippo–YAP pathway. SC (30 μg/mL) induced the phosphorylation of YAP, a key target of the Hippo–YAP pathway, in HepG2 (Figure 5A), Hep3B (Figure 5B), and Huh7 (Figure 5C) cells. The maximum phosphorylation of YAP was observed at 10 min–3 h in HepG2 cells. LATS1, an upstream inhibitory factor of YAP, was also phosphorylated by the SC treatment in HepG2 (Figure 5A), Hep3B (Figure 5B), and Huh7 (Figure 5C) cells. In addition, the link between the activation of the LKB1-AMPK pathway and the activation of the Hippo–YAP pathway by the SC treatment was investigated. SC (30 μg/mL) induced the phosphorylation of LKB1, AMPK, and LATS1 in HepG2 cells but not in LKB1-deficient HeLa cells (Figure 5D).

### 2.6. Procyanidin B2 in SC Protects Hepatocytes

**Figure 6:**
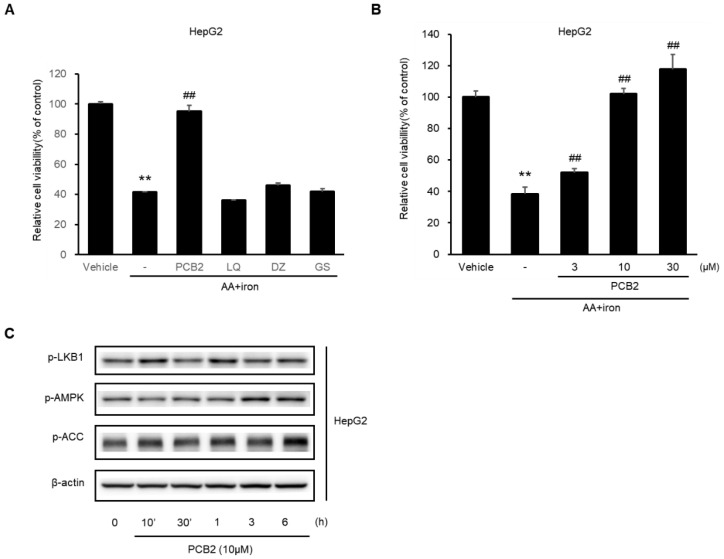
Effects of Procyanidin B2, a representative component of SC. (**A**) HepG2 cells were treated with 10 μM PCB2, 30 μM LQ, DZ, and GS for 1 h and then incubated with 10 μM AA plus some drugs for 12 h, followed by incubation with 5 μM iron for 6 h. The cell viability was assessed using an MTT assay. (**B**) HepG2 cells were treated with PCB2 (3, 10, 30 μM) for 1 h, and then proceeded sequentially as described in (**A**). The data represent the mean ± SD (** *p* < 0.01 between the control and AA + iron-treated cells; ## *p* < 0.01 between AA + iron and AA + iron-treated cells with compounds). (**C**) Immunoblotting analysis to verify the expression of the AMPK signaling pathway was performed with HepG2 cell lysates. The cells were incubated in serum-free media for 12 h, followed by treatment with 10 μM PCB2 for the indicated time.

The cytoprotective effects of the representative components (procyanidin B2, liquiritigenin, daidzein, and genistein) in SC were investigated. Treatment with 10 μM of procyanidin B2 (PCB2) protected hepatocytes from AA + iron-induced cytotoxicity. In contrast, liquiritigenin (LQ), daidzein (DZ), and genistein (GS) were ineffective (Figure 6A). PCB2 significantly inhibited AA + iron-induced apoptosis in a dose-dependent manner (Figure 6B). PCB2 induced the phosphorylation of LKB1, AMPK, and ACC, which are critical factors of the LKB1-AMPK pathway (Figure 6C). These data suggest that PCB2 protects hepatocytes by mediating the LKB1-AMPK signaling pathway.

### 2.7. SC Ameliorates APAP-Induced Acute Liver Injury

**Figure 7:**
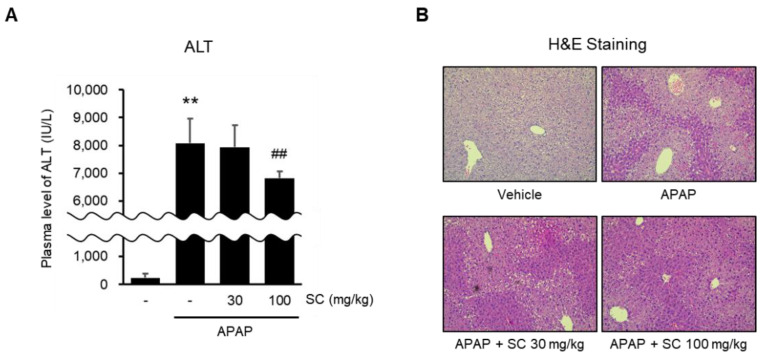
Effects of SC on APAP-induced liver injury and AMPK activation in mice. After the oral administration of 100 mg/kg SC to mice for three consecutive days, they were given a fasting time of 16 h. Subsequently, 500 mg/kg APAP was injected orally. (**A**) Serum ALT (alanine aminotransferase) levels in mice revealed changes in liver injury. The data represent the mean ± SD (** *p* < 0.01 between the vehicle control; ## *p* < 0.01 between APAP treatments). (**B**) Histochemical analysis of liver tissue in mice was performed by H&E staining (×200).

AMPK activation by SC protects hepatocytes in vitro. Therefore, the effects of SC on an APAP-induced liver injury mice model were investigated. An oral injection of APAP (500 mg/kg) increased the serum ALT levels. On the other hand, oral administration of 100 mg/kg SC for three days before the APAP injection reduced the serum ALT levels significantly (Figure 7A). The histopathological changes were observed in the liver by H&E staining. Pretreatment of 100 mg/kg SC for three days attenuated the morphological changes of the liver induced by the APAP treatment (Figure 7B).

## 3. Discussion

Drugs and therapeutic candidates with cytoprotective effects have been reported to inhibit oxidative stress via AMPK activation and the induction of antioxidant enzymes [26]. Rifampicin, an antibiotic, activates the LKB1-AMPK pathway to suppress oxidative stress and mitochondrial damage in liver cells [27]. Metformin, a medication for diabetes, has been reported to prevent vascular damage by inhibiting endoplasmic reticulum (ER) stress through AMPK activation [28]. *Rhizoma Coptidis*, a medicinal herb, and its bioactive component, berberine, have pharmaceutical potential for aging-related diseases by anti-oxidation and AMPK activation [29]. Activation of the LKB1-AMPK pathway by *Liqustri lucidi Fructus* protected hepatocytes and alleviated acute hepatic injury in an in vivo model [30]. In the present study, SC protected hepatocytes against oxidative stress induced by AA + iron through mediation with the AMPK signaling pathway in vivo and in vitro.

The Hippo–Yap pathway is another crucial regulator in cell protection. Upon activation of the Hippo signaling pathway, MST1/2 and LATS1/2 included in the Hippo pathway kinase cascade are phosphorylated, which directly phosphorylates and inactivates YAP and TAZ [31,32]. The inactivation of YAP/TAZ interferes with its localization into the cell and inhibits the formation of a complex with TEAD, a YAP target transcription factor. As a result, the inhibition of YAP/TAZ and TEAD complex formation does not lead to the expression of the target genes that regulate the cell cycle, cell migration, and cell fate [31,32]. It has also been shown that AMPK-dependent LATS phosphorylation induced YAP phosphorylation, and AMPK sometimes directly phosphorylates the YAP Ser94 residue, the site of interaction with TEAD [33,34]. In this study, SC regulated the Yap signals under the AMPK pathway, which helps protect the hepatocytes against oxidative stress induced by AA + iron.

In the cell assays, the AA + iron model was enrolled to test the effects of SC on oxidative stress. AA increases the production of pro-inflammatory eicosanoids and impairs the regulation of hepatic and adipose function, predisposing to non-alcoholic fatty liver disease (NAFLD) [35]. In hepatocytes, a high ratio of omega-6 fatty acids (arachidonic acid, AA) to omega-3 fatty acids may promote the lipogenic pathways and induce high levels of ROS production, which can lead to mitochondrial dysfunction [36]. The excessive production of intracellular ROS activates phospholipase A2, which hydrolyzes phospholipase and fatty acids that form the cell membrane, and results in the release of AA [37]. The isolated AA increases ROS, which is linked to impaired mitochondrial respiratory activity [38,39]. Consequently, excessive ROS causes liver disease and activates hepatic stellate cells, which are one of the keys to liver injury and fibrogenesis [40].

Iron is another key component of oxygen transport and storage and is stored in the liver, muscles, spleen, and bone marrow. On the other hand, excess iron deposition generates ROS, which leads to liver injury and hepatic fibrosis through several mechanisms [41]. In addition, iron overload enhances AA release and eicosanoid production in cardiomyocytes and induces cardiomyopathy [42]. A combined treatment of AA and iron, which induces synergistic cytotoxicity, is considered a model of oxidative stress-induced hepatocellular injury [43,44]. In previous studies applying the AA and iron model, AA + iron-induced cytotoxicity led to ROS generation, GSH reduction, and mitochondrial dysfunction in hepatocytes [45,46].

In this study, PCB2 was active in the aspect of the antioxidant effects of SC. PCB2 contained in grape seeds, apples, and cacao beans has been reported to be the major bioactive component of SC [47]. Previous studies showed that PCB2 had anti-inflammatory effects on vascular endothelial cells, inhibited neurological deficits and blood–brain barrier disruption, and alleviated chronic nonbacterial prostatitis [48,49,50]. PCB2 attenuates colitis-associated cancer formation by inhibiting oxidative stress and diet-induced hepatic steatosis [51,52]. In particular, it protects hepatocytes against CCl_4_-induced acute hepatotoxicity [53].

In in vitro experiments, we applied HepG2, Hep3B, and Huh7 among hepatocellular carcinoma (HCC) cell lines derived from humans. These three kinds of cell lines represent epithelial features and well-differentiated HCC in the early phases [54,55,56]. Using a line of cells, it is possible to observe variously the expression level and time of major signal transduction proteins. In particular, it was observed that the activation time of the molecules by the drug was slightly different in signal transduction, including AMPK. The effects of SC and PCB2 in the cells derived from other animals need to be confirmed in the future.

In animals, the acute hepatic damage model induced by APAP was used. The annual incidence of acute liver failure is approximately 5.5 per million of the population, and most acute liver failure cases are drug-induced hepatotoxicity [57]. In particular, intentional or unintentional acetaminophen overdose explained 46% of cases in the United States [58]. Although acute liver failure is uncommon, interest and study continue in multiple disciplines because it affects all organ systems and has a mortality rate of 30% [59].

The mechanism of APAP-induced liver cell death is the release of intermembrane proteins, such as endonuclease G, which induces DNA fragmentation via the induction of mitochondrial membrane permeability transition caused by enhanced oxidative stress [60]. Therefore, APAP is important in drug-induced hepatotoxicity as assessed by disruption of the cellular defense system and induction of mitochondrial dysfunction [61,62]. In this study, SC inhibited APAP-induced liver failure, which means SC has protective effects in vivo.

## 4. Materials and Methods

### 4.1. Chemicals and Reagents

Anti-phospho-acetyl-CoA carboxylase (ACC), anti-phospho-AMPKα, anti-phospho-LKB1, anti-Phospho-LATS1, anti-phospho-YAP, anti-YAP, anti-Poly (ADP-ribose) polymerases (PARP), anti-caspase-3, anti-B-cell lymphoma-extra-large (Bcl-xL), and anti-β-actin were purchased from Cell Signaling Technology (Danvers, MA, USA). HRP-conjugated anti-rabbit IgG and HRP-conjugated anti-mouse IgG were bought from Enzo Life Sciences (Farmingdale, NY, USA). Polyethylene glycol (PEG) 400 is manufactured by Yakury Pure Chemical Co., Ltd. (Kyoto, Japan). SC was produced by water extraction of the medicinal herb, *Spatholobi Caulis* (Daewon pharmacy, Daewon, Korea) as previously described [17,19]; 3-(4,5-dimethylthiazol-2-yl)-2,5-diphenyl-tetrazolium bromide (MTT), rhodamine 123 (Rh123), 2′,7′-dichlorofluorescein diacetate (DCFH-DA), arachidonic acid (AA), ferric nitrilotriacetic acid (iron), Harris hematoxylin and eosin (H&E), and other reagents were purchased from Sigma-Aldrich (St. Louis, MO, USA).

### 4.2. Cell Lines and Cell Culture

HepG2, Hep3B, and HeLa cells (American Type Culture Collection, Rockville, MD, USA) were maintained in Dulbecco’s modified Eagle’s medium (DMEM) high glucose with 10% fetal bovine serum (FBS), 50 μg/mL streptomycin, and 50 units/mL penicillin, as previously described [17,19]. Huh-7 cells were cultured in RPMI 1640 containing 10% FBS, 50 μg/mL streptomycin, and 50 units/mL penicillin.

### 4.3. Cell Viability Assay

In 48-well plates, HepG2 cells were plated in each well at a density of 1 × 10^5^ for 24 h and then incubated in free MEM medium without FBS for 12 h. Cells were incubated with 3, 10, 30 μg/mL SC for 1 h and then treated with 10 μM AA for 12 h, followed by 5 μM iron for 6 h as previously described [17,19].

### 4.4. Measurement of Reactive Oxygen Species (ROS) Production

DCFH-DA, cell-permeant reagent fluorogenic dye, is an indicator of ROS activity in cells. After uptake into cells, DCFH-DA is deacetylated to non-fluorescent compounds by intracellular esterases, which are subsequently oxidized by ROS to turn into highly fluorescent 2′,7′-dichlorofluorescein (DCF). In 96-well black plates, HepG2 cells were plated in each well at a density of 1 × 10^4^ for 24 h. After 12 h of incubation in a FBS-free MEM medium, treatment of 30 μg/mL SC, 10 μM AA, and 5 μM iron for 1 h. Subsequently, cells were incubated with 10 μM DCFH-DA at 37 °C for 1 h. DCF fluorescence was detected by an ELISA microplate reader.

### 4.5. Measurement of Mitochondrial Membrane Potential

Mitochondrial membrane potential (MMP) was measured by flow cytometry with Rhodamine 123 (Rh123), a cell-permeant, cationic, and green-fluorescent dye, as previously described [17,19]. HepG2 cells were plated at a density of 1 × 10^5^ cells per well for 24 h and then incubated in free MEM medium without FBS for 12 h. Cells were incubated with 30 μg/mL SC for 1 h and then treated with 10 μM AA for 12 h, followed by 5 μM iron for 1 h. After staining the cells with 0.05 μg/mL Rh123 for 1 h, they were harvested by Trypsin. MMP changes were monitored by recording 10,000 events in cells.

### 4.6. Immunoblot Analysis

Cell lysates were prepared using RIPA buffer at 4 °C according to the previously described protocol [63]. Bands were developed using the enhanced chemiluminescence reagent and Chemidoc image analyzer (Vilber Lourmat, France).

### 4.7. Animals and Treatment

The procedures for animal experiments were approved and monitored by the Institutional Animal Care and Use Committee of Dongguk University. Male C57BL/6 mice (6 weeks, 20–21 g) were purchased from Charles River Orient Bio (Seongnam, Korea). Mice were randomly divided into 4 groups: vehicle-treated control, APAP, APAP + SC 30 mg/kg, and APAP + SC 100 mg/kg. Mice were orally administrated with SC (30 mg/kg or 100 mg/kg, dissolved in 40% PEG) or the vehicle (40% PEG). After oral treatment for 3 consecutive days, a fasting time was given for 16 h. A total of 500 mg/kg APAP (in 40% PEG) was orally injected, and mice were sacrificed at 6 h after APAP [17].

### 4.8. Blood Chemistry

Aspartate aminotransferase (ALT) in serum was analyzed by the Chaon Research Lab (Seongnam, Korea) using ALT ELISA kits (Chema Diagnostica, Monsano, AN, Italy) and the AU680 chemistry analyzer (Beckman Coulter, CA, USA).

### 4.9. Histopathology

Liver tissue separated from the left lateral lobe was fixed in neutral buffered 4% formaldehyde. Sectioned liver tissues were stained with hematoxylin and eosin (H&E) and observed with light microscopy (Nikon, Tokyo, Japan).

### 4.10. Statistical Analysis

To verify significant differences between treatment groups, a Student’s *t*-test analysis or ANOVA was performed. Blood chemistry and histomorphometric values, as well as data from animal experiments, are presented as mean ± SD. The criterion of statistical significance was established as *p* < 0.05 or *p* < 0.01.

## 5. Conclusions

In conclusion, SC protects hepatocytes against AA + iron-induced cytotoxicity and inhibits ROS generation and mitochondrial dysfunction via activation of the LKB1-AMPK pathway and Hippo–Yap pathway in vitro. PCB2, a representative component of SC, inhibited cytotoxicity and activated the LKB1-AMPK pathway, a key bioactive component. In mice, oral injection of SC ameliorates APAP-induced liver injury and phosphorylates AMPK in the liver. These results reveal the potential of SC and key bioactive components as candidates for hepatoprotection via antioxidants.

## Data Availability

The data presented in this study are available on request from the corresponding author.
